# A protospacer adjacent motif‐free, multiplexed, and quantitative nucleic acid detection platform with barcode‐based Cas12a activity

**DOI:** 10.1002/mco2.310

**Published:** 2023-07-02

**Authors:** Miaojin Zhou, Chunhua Zhang, Miaomiao Chen, Zhiqing Hu, Menglin Li, Zhuo Li, Lingqian Wu, Desheng Liang

**Affiliations:** ^1^ Center for Medical Genetics & Hunan Key Laboratory of Medical Genetics School of Life Sciences Central South University Changsha Hunan China; ^2^ Department of Medical Genetics Yunnan Maternal and Child Health Care Hospital Kunming Yunnan China

**Keywords:** barcode‐based Cas12a, copy number variation, simultaneous detection of multiple targets, spinal muscular atrophy, universal PAM and crRNA

## Abstract

Clustered regularly interspaced short palindromic repeat (CRISPR)‐based biosensors have been developed to facilitate the rapid and sensitive detection of nucleic acids. However, most approaches using CRISPR‐based detection have disadvantages associated with the limitations of CRISPR RNA (crRNA), protospacer adjacent motif (PAM) or protospacer flanking sequence restriction, single channel detection, and difficulty in quantitative detection resulting in only some target sites being detected qualitatively. Here, we aimed to develop a barcode‐based Cas12a‐mediated DNA detection (BCDetection) strategy, which overcomes the aforementioned drawbacks and enables (1) detection with a universal PAM and crRNA without PAM or crRNA restriction, (2) simultaneous detection of multiple targets in a single reaction, and (3) quantitative detection, which can significantly distinguish copy number differences up to as low as a two‐fold limit. We could efficiently and simultaneously detect three β‐thalassemia mutations in a single reaction using BCDetection. Notably, samples from normal individuals, spinal muscular atrophy (SMA) carriers, and SMA patients were significantly and accurately distinguished using the quantitative detection ability of BCDetection, indicating its potential application in β‐thalassemia and SMA carrier screening. Therefore, our findings demonstrate that BCDetection provides a new platform for accurate and efficient quantitative detection using CRISPR/Cas12a, highlighting its bioanalytical applications.

## Graphical abstract

We developed a barcode‐based Cas12a‐mediated DNA detection (BCDetection) strategy. With efficient transformation and amplification of hybridization signals to fluorescence signals, BCDetection not only enables detection of multiple targets in a single reaction without intrinsic PAM or crRNA restriction, but also can significantly distinguish copy number differences up to as low as a twofold limit.

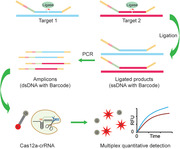

## 1 INTRODUCTION

Rapid, accurate, economic, and sensitive detection of nucleic acids plays an important role in the early and rapid diagnosis of infectious diseases, screening and diagnosis of genetic diseases, and detection of foodborne pathogens. RNA‐guided clustered regularly interspaced short palindromic repeat (CRISPR) and CRISPR‐associated (CRISPR/Cas) detection platforms, including SHERLOCK based on Cas13,^1^, ^2^ DETECTR^3^ or HOLMES^4^ based on Cas12a, and CDetection based on Cas12b,^5^ with high sensitivity and specificity have recently been developed. The key principle of the CRISPR nucleic acid detection system is *trans*‐cleavage, also known as acquired collateral cleavage activity.^5^, ^6^ The Cas protein can specifically recognize and cleave the target DNA/RNA under the guidance of a CRISPR RNA (crRNA) with high sensitivity, triggering the collateral cleavage activity of Cas to nonspecific cleavage of the ambient single‐stranded DNA (ssDNA) or RNA reporter.

CRISPR‐based detection methods have been established and broadly used for nucleic acid detection. The SHERLOCK platform can detect RNA, such as that in SARS‐CoV‐2, via T7 transcription in vitro followed by activated Cas13‐based noncanonical *trans*‐cleavage of the RNA reporter.^7^, ^8^ By recognizing the target double‐stranded DNA (dsDNA) sequence containing a T‐rich protospacer adjacent motif (PAM), specifically activated Cas12a or Cas12b protein can cleave the target dsDNA and then nonspecifically cleave the ssDNA reporter.^5^, ^6^

Previously, our group and other researchers have used these technologies for infectious disease detection^9^, ^10^, ^11^ as well as for germline and somatic mutation genotyping.^10^, ^12^, ^13^, ^14^ However, these technologies have several limitations, including PAM or protospacer flanking sequence (PFS) restriction, single channel detection (only one target molecule can be distinguished in a single reaction), and difficulty in quantitative detection. Gootenberg et al.^15^ established a multiplex detection platform for detection of four targets in a single reaction, which relies on different reporters, four guide crRNAs, and Cas enzymes, including LwaCas13a, PsmCas13b, CcaCas13b, and AsCas12a. Moreover, the platform has the PAM/PFS sequence restriction.^15^ Luo et al.^16^ combined the droplet digital reverse transcription loop‐mediated isothermal amplification and CRISPR/Cas12b detection system to realize quantitative detection of viral RNA, this detection method requires bulky equipment and trained operators.

In this study, we aimed to develop a barcode‐based Cas12a‐mediated DNA detection (BCDetection) strategy to overcome these limitations, wherein the recognized sequences for CRISPR RNA (crRNA) were integrated into the probe, named barcode. Herein, target recognition was achieved via probe hybridization rather than direct crRNA binding. Cas12a‐based universal detection can be accomplished via probe hybridization with specific target sequences, without the requirement for intrinsic PAMs. Moreover, probe hybridization enabled the creation of a multiplex platform using multiple pairs of probes. The detection efficiency of BCDetection was demonstrated by the detection of beta‐globin gene (*HBB*) mutations at multiple sites in a single reaction and quantitatively profiling the copy number of the survival motor neuron 1 gene (*SMN1*). Our findings demonstrate that BCDetection system is a new powerful technique for PAM‐free, multiplexed, and quantitative nucleic acid detection.

## 2 RESULTS

### 2.1 Construction of the primer‐based Cas12a detection platform

**FIGURE 1:**
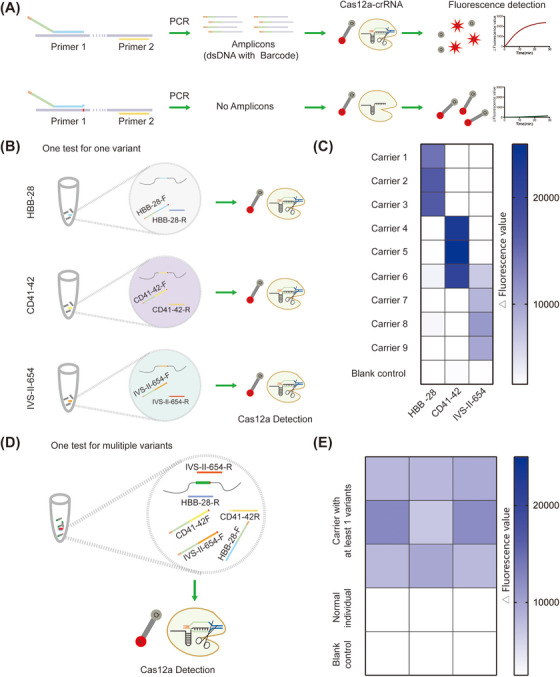
The primer‐based Cas12a detection system (PCDetection) for β‐thalassemia mutation detection. (A) Schematic diagram representing PCDetection. Primer 1 contains protospacer adjacent motif (PAM) and CRISPR RNA (crRNA) sequences (named Barcode), and the base located in the 3′‐end is modified with locked nucleic acid, which can increase the annealing temperature. When the template and primer binding sequences completely match, an amplicon with the Barcode is generated and detected by Cas12a, then yields fluorescence signals by cleaving ssDNA‐fluorophore‐quencher (FQ) probes. If there is a mismatch between the template and the3′‐end of primer binding sequence, there will be no amplification product and no fluorescence signal. (B) Schematic diagram representing detection of one variant of β‐thalassemia dominant mutation, HBB‐28, CD41‐42, and IVS‐II‐654, using PCDetection in a single test. The circles represent the components of the system. (C) Nine β‐thalassemia carriers were detected using specific PCDetection for the three β‐thalassemia mutations, HBB‐28, CD41‐42, and IVS‐II‐654. Each assay was performed in triplicates. (D) Schematic diagram representing detection of multiple variants of β‐thalassemia mutation, HBB‐28, CD41‐42, and IVS‐II‐654, using PCDetection in a single test. The circle represents the components of the system (E) In‐sample multiplexed detection of β‐thalassemia mutations, HBB‐28, CD41‐42, and IVS‐II‐654, with Cas12a. Each assay was performed in triplicates.

To achieve PAM‐free detection, we designed a primer with a universal crRNA and PAM sequence. The scheme of the primer‐based Cas12a detection (PCDetection) system was presented (Figure 1A). In this assay system, the target sequence could be specifically amplified using specific Primer‐1 and Primer‐2. The amplified products contained a barcode that could activate the collateral cleavage activity of Cas12a and yield fluorescence signals by cleaving ssDNA‐fluorophore‐quencher (FQ) probes. The PCDetection system was designed and constructed. The genomic DNA (gDNA) samples containing three β‐thalassemia mutations, *HBB*:c.‐78A>G (HBB‐28), *HBB*: c.126_129delCTTT (CD41‐42), and *HBB*:c.316‐197C>T (IVS‐II‐654), were used to evaluate the detection performance of the PCDetection system. The samples of carriers with mutations and normal individuals could be significantly distinguished (Figures 1B and C), which was consistent with clinical examination, suggesting that PCDetection can effectively detect the target sequence without requiring intrinsic PAMs around the target sequence. Thus, the use of PCDetection will enable to extend the scope of testing, especially for specific variants in genetic disease, and provide another approach to improve the accuracy of detection through optimization of primers and conditions, not just crRNAs.

In many situations, the detection of multiple target molecules in a single reaction is required. Therefore, we evaluated the multiplexing potential of the PCDetection platform. We designed three pairs of primers, HBB‐28‐F/HBB‐28‐R, CD41‐42‐F/ CD41‐42‐R, and IVS‐II‐654‐F/ IVS‐II‐654‐R, wherein all forward primers contained the same barcode. The primers for the three mutated loci were mixed to amplify the gDNA of carriers and normal participants by multiplex PCR, and ddH_2_O was used as a blank control. Amplicons were detected using Cas12a. PCDetection was able to distinguish between the samples of carriers and normal individuals, indicating that the carriers harbored at least one of the HBB‐28, CD41‐42, or IVS‐II‐654 mutations (Figures 1D and E). These results suggest that PCDetection can simultaneously detect multiple target sequences, which are useful for the detection of genetic variants and pathogens.

### 2.2 Quantitative detection capability of Cas12a

**FIGURE 2:**
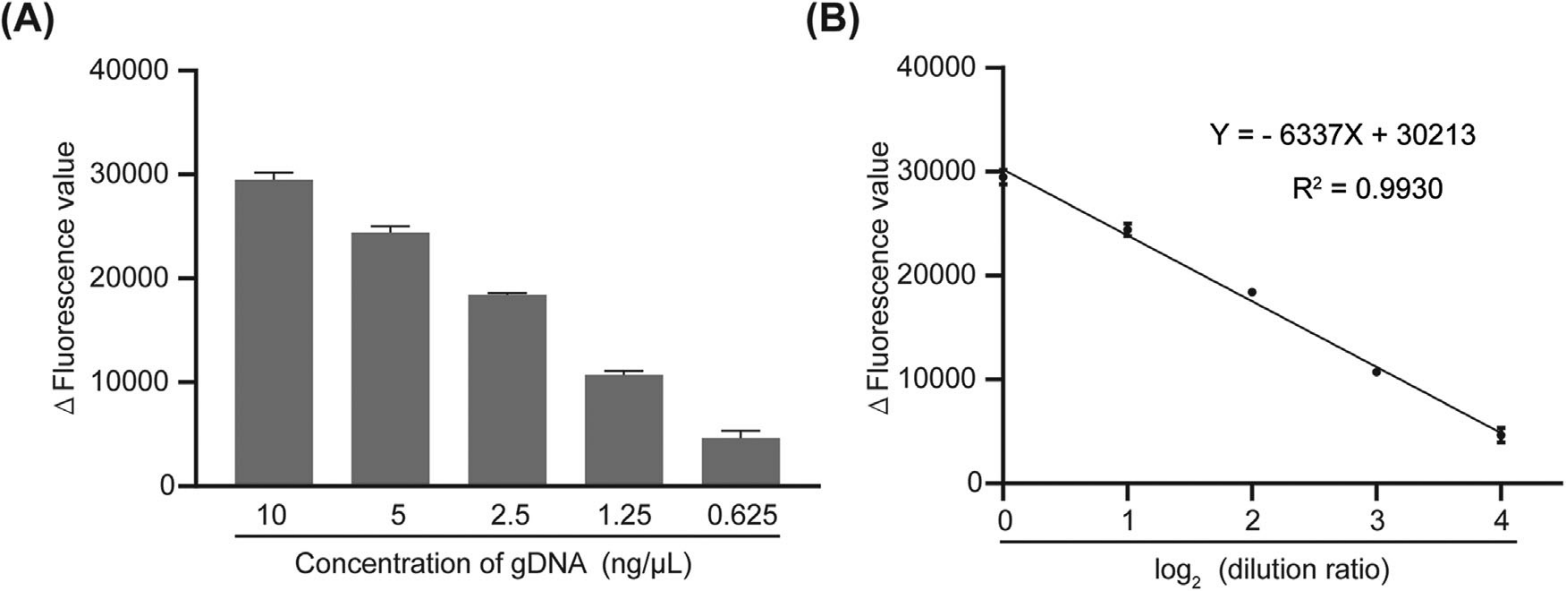
Quantitative detection capability of Cas12a. (A) The fluorescence values of the sequentially diluted genomic DNA (gDNA) samples. (B) The linear relationship between gDNA sample concentration and fluorescence value. *Δ*, represents subtracted the background fluorescence. Error bars represent the means ± SD; *n* = 3.

Quantitative detection of the copy numbers of target genes or pathogens plays a crucial role in medical science, including assessing the risk of bearing children with genetic disease, estimating health risks, and classifying disease severity. To verify whether the Cas12a detection system has the capability of quantitative detection, we sequentially diluted a sample of normal human gDNA (10 ng/μL) to 5, 2.5, 1.25, and 0.625 ng/μL concentrations. After PCR amplification, the amplicons were detected using SMA‐Cas12a, which was established previously.^17^ The fluorescence values gradually decreased with the decreasing gDNA concentration (Figure 2A). Linear regression analysis was performed using log_2_(dilution ratio) as the independent variable (*X*) and the fluorescence value as the dependent variable (*Y*). The fluorescence values were negatively correlated with log_2_(dilution ratio) *Y* = −6337*X* + 30213, *R* ^2^ = 0.9930 (Figure 2B). Gootenberg et al.^15^ and Li et al.^18^ used Cas13a and Cas12b to detect nucleic acid molecules with different copy numbers and demonstrated that their technique could distinguish the samples at a 10‐fold copy number difference. In this study, we confirmed the quantitative detection ability of Cas12a and found that even a two‐fold difference in copy number could be effectively detected, indicating that copy number variation (CNV) in human genome could be detected, for instance, the difference between SMA carriers and normal individuals.

### 2.3 Establishment of BCDetection detection platform

**FIGURE 3:**
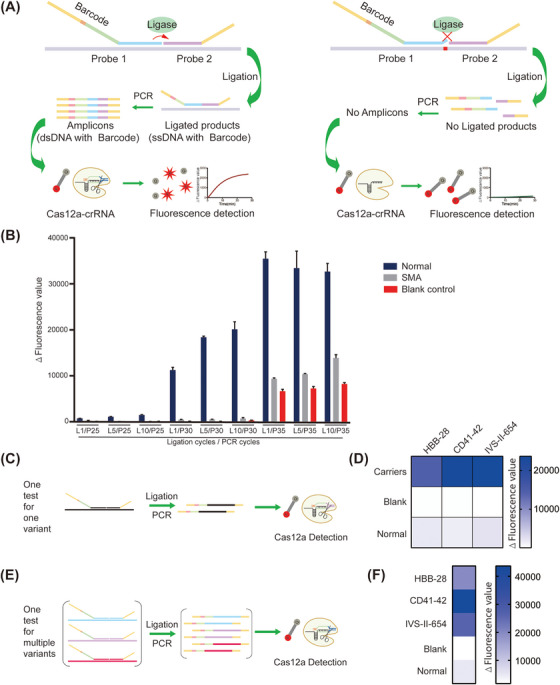
The barcode‐based Cas12a detection system (BCDetection) used for detecting β‐thalassemia mutations. (A) Flow chart representing BCDetection workflow. Probe 1 contains a barcode sequence, including the PAM and crRNA sequences, Primer F sequence, and specific hybridized sequence. Probe 2 contains the Primer R binding sequence and specific hybridized sequence. When the template and probe binding sequence completely match, a ligated product with the targeted sequence of crRNA is generated by Primer F/R and detected by Cas12a, then yields fluorescence signals by cleaving ssDNA‐FQ probes. When there is a mismatch between the template and the 3′‐end of Probe 1 binding sequence, no ligated product is generated, and no fluorescence signal. (B) Cas12a fluorescence assay was used to detect the products generated under different reaction conditions for ligation and PCR amplification. L, ligation cycles; P, PCR cycles. *Δ*, represents subtracted the background fluorescence. (C) Schematic diagram representing detection of one variant of β‐thalassemia mutation using BCDetection in a single test. (D) Three β‐thalassemia carriers were detected using specific BCDetection for the three β‐thalassemia mutations, HBB‐28, CD41‐42, and IVS‐II‐654. Each assay was performed in triplicates. *Δ*, represents subtracted the background fluorescence. (E) Schematic diagram representing detection of multiple variants of β‐thalassemia mutations using BCDetection in a single test. (F) In‐sample multiplexed detection of β‐thalassemia mutations (HBB‐28, CD41‐42, and IVS‐II‐654) using BCDetection. Each assay was performed in triplicates. *Δ*, represents subtracted the background fluorescence.

To overcome the problem of the accuracy of qualitative assessment, we optimized the PCDetection system and established a BCDetection platform, with a pair of probes containing universal crRNA, PAM sequence, and primer binding site (called barcode). The principle of BCDetection was illustrated (Figure 3A). The hybridization P1 was designed to have a 3′‐end specific for the target sequence so that it did not ligate with the other hybridization P2 when P1 was hybridized with nontargeted sequences, even if the target sequence differed only by a single‐nucleotide in the P1 at the 3′‐end. Hybridization of the two probes to the target would bring the two probes into proximity, and the ligase would catalyze the ligation of the two probes into ssDNA. In the presence of primer F/R and polymerase, the ligation products would be amplified into dsDNA products that could be recognized by a universal crRNA. Then, the trans‐cleavage ssDNA activity of Cas12a would be activated, thereby releasing fluorescence.

To verify the principle of BCDetection and to explore the appropriate reaction conditions, the number of ligation cycles and PCR amplification cycles were optimized. We designed P1/P2 targeting c.840 of *SMN1*, which is a hotspot pathogenic mutation in SMA. *SMN2*, a gene paralogous to *SMN1*, has a single‐nucleotide variation at c.840. At least one copy of the *SMN2* gene is present in almost all SMA patients.

The fluorescence obtained for normal individuals significantly increased upon increasing the number of PCR amplification cycles; for 30 cycles of PCR amplification, the difference in fluorescence signals between normal individuals and SMA patients was more than 24.73‐fold (Figure 3B). In particular, for five ligation cycles, the difference in fluorescence signals between normal individuals and SMA patients reached 35.39‐fold (Figure 3B). These data indicate that BCDetection is sensitive to the presence of mismatches near the ligation site and can effectively translate the information of the target sequence to the products of probe ligation for subsequent PCR and Cas12a‐mediated detection.

Next, we examined whether BCDetection could effectively detect multiple target molecules in a single reaction. Three pairs of probes, HBB‐28‐P1/HBB‐28‐P2, CD41‐42‐P1/CD41‐42‐P2, and IVS‐II‐654‐P1/IVS‐II‐654‐P2, were designed; the P1s had the same barcode. The samples of carriers with mutations and normal individuals could be significantly distinguished by BCDetection in one test for one variant system (Figures 3C and D). All the probes were mixed with the gDNA sample, and the fluorescence signals were measured. BCDetection showed the same ability as that of PCDetection to distinguish between carriers and normal controls (Figures 3E and F). Together, these results indicate that BCDetection has the capacity to detect multiple target sequences concurrently.

### 2.4 Quantitative detection capability of BCDetection

**FIGURE 4:**
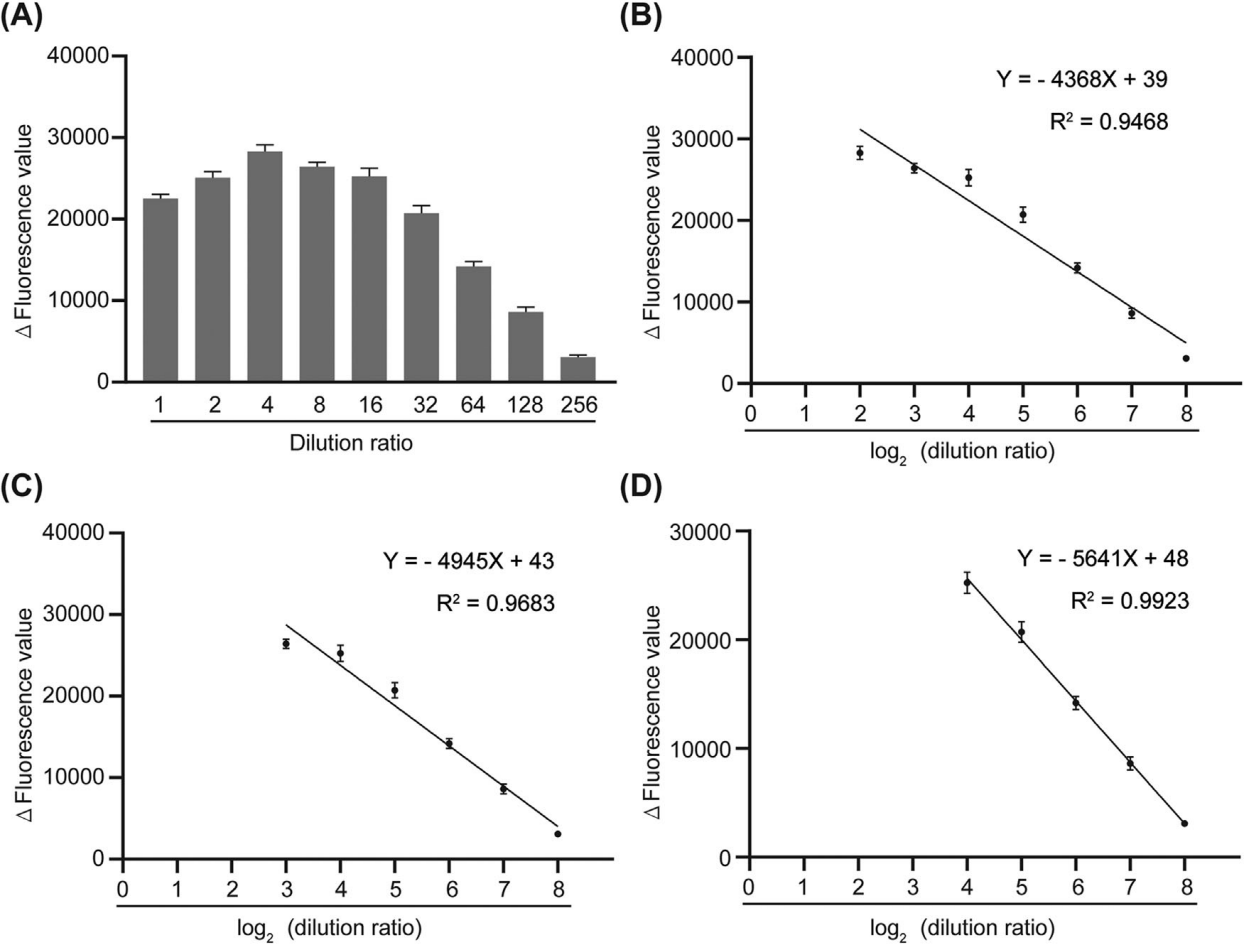
Quantitative detection capability of barcode‐based Cas12a detection system. (A) The fluorescence values of the sequentially diluted genomic DNA (gDNA) samples. (B) The linear relationship between the concentration of gDNA samples and fluorescence value in the range of 4–256‐fold dilutions. (B) The linear relationship between the gDNA concentration and fluorescence value in the range of 8–256‐fold dilutions. (D) The linear relationship between the concentration of gDNA samples and fluorescence value in the 16–256‐fold dilution range. Error bars represent the means ± SD; *n* = 3. *Δ*, represents subtracted the background fluorescence.

Another goal of BCDetection was to address the accuracy of quantitative detection. To verify the quantitative detection ability of BCDetection, a normal individual gDNA sample (50 ng/μL) was diluted, and gDNA samples with seven concentration gradients were obtained by doubling dilution. After hybridization, ligation, and PCR amplification reaction, the seven concentration gradients of amplification products were detected by BCDetection (Figure 4A). Fluorescence values were negatively correlated with log_2_(dilution ratio) in the range of 4–256 folds dilution, *Y* = −4368*X* + 39, *R*2= 0.9468 (Figure 4B). In the range of 8–64 folds dilution, *Y* = −4945*X* + 43, *R* ^2^ = 0.9683 (Figure 4C), and in the range of 16–256 folds dilution, *Y* = −5641*X* + 48, *R*2= 0.9923 (Figure 4D). These results demonstrate that BCDetection can be used for quantitative detection.

### 2.5 Quantitative detection using BCDetection

**FIGURE 5:**
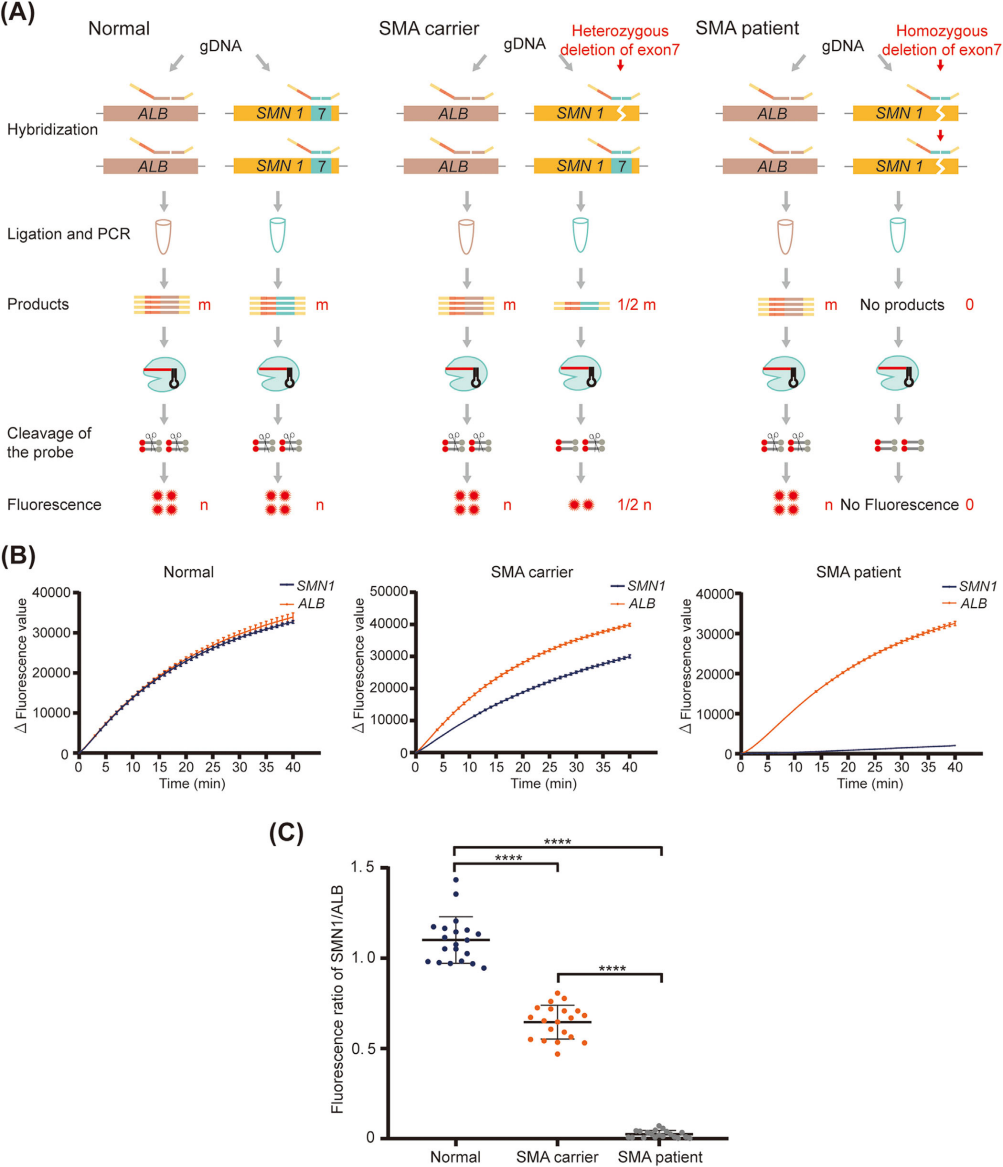
The two‐tube barcode‐based Cas12a detection (BCDetection) method used for survival motor neuron 1 gene (*SMN1*) copy number detection. (A) Schematic diagram representing *SMN* copy number detection. Theoretically, for normal individuals carrying two copies of *SMN1* and albumin (*ALB*), after hybridization, ligation, and amplification, the copy number of amplicons of the two genes is approximately the same; furthermore, the final ratio of increment in fluorescence value of *SMN1* to that of *ALB* is close to 1. In spinal muscular atrophy (SMA) carriers, the ratio is 0.5. In SMA patients, the ratio is near 0. (B) The change curves of fluorescence value obtained from normal individuals, SMA carriers, and SMA patients using BCDetection. *Δ*, represents subtracted the background fluorescence. Error bars indicate mean ± SEM; *n* = 3. (C) Comparison of the ratio of increment in *SMN1* fluorescence value to *ALB* fluorescence value in normal individuals, SMA carriers, and SMA patients detected using BCDetection. Error bars indicate means ± SD; *n* = 20. ****, *p* < 0.0001.

To eliminate the interference of sample gDNA initial concentration in quantitative detection, relative quantitative detection of *SMN1* exon 7 copy number was performed with the *ALB* as an internal reference gene. The SMN1‐probes (SMN‐P1/P2) were targeted against *SMN1* c.840, and ALB‐probes (ALB‐P1/P2) were targeted against *ALB*. The products of the SMN1‐probe had the same PCR primer binding sequence and crRNA recognition sequence as those of the products of the ALB‐probe. In principle, for normal individuals harboring two copies of *SMN1* and *ALB*, the copy number of the amplified products of the two genes is approximately the same after ligation and amplification. Theoretically, Cas12a detection showed that the ratio of the fluorescence signal of *SMN1* to that of *ALB* was close to 1. For SMA carriers, the ratio was nearly 0.5, and for SMA patients, it tended to 0 (Figure 5A). To evaluate the feasibility of the BCDetection assay in the diagnosis of clinical samples, we analyzed gDNA isolated from normal individuals, SMA carriers, and SMA patients. *SMN1* and *ALB* were detected in separate reactions, and this method was called the two‐tube method. The fluorescence curves of *SMN1* exon 7 and *ALB* were almost identical, suggesting that the copy number of *SMN1* exon 7 was the same as that of *ALB* in normal participants. The fluorescence value curves of *SMN1* exon 7 were always lower than those of *ALB*, indicating that the copy number of *SMN1* exon 7 was lower than that of *ALB* in SMA carriers. For SMA patients, the fluorescence value curve of *SMN1* exon 7 was near the baseline, whereas that of ALB was comparatively high (Figure 5B). We detected 60 clinical samples, including 20 samples from normal individuals, 20 samples from SMA carriers, and 20 samples from SMA patients. The results showed that the ratio of fluorescence signals was notably distinguished among the normal participants (1.10 ± 0.13), SMA carriers (0.65 ± 0.09), and SMA patients (0.03 ± 0.02) (Figure 5C). Therefore, these results demonstrated the feasibility of using the BCDetection assay for distinguishing CNV (two‐tube method) in clinical diagnosis.

**FIGURE 6:**
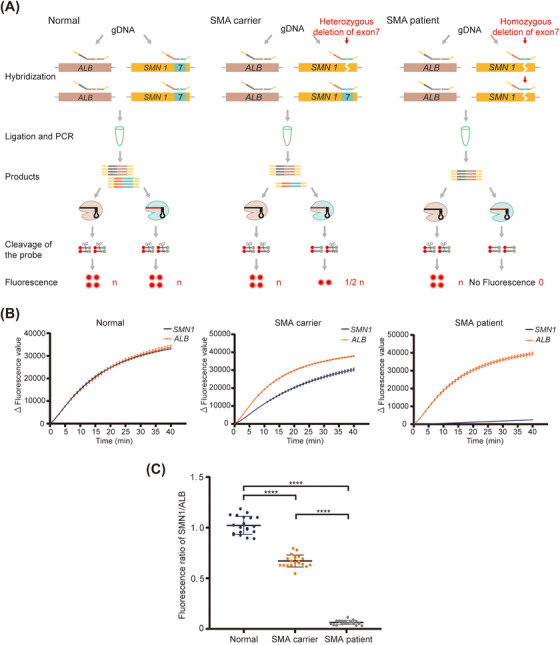
The one‐tube barcode‐based Cas12a detection (BCDetection) method used for survival motor neuron 1 gene (*SMN1*) copy number detection. (A) Schematic diagram representing SMN copy number detection using the one‐tube BCDetection method. After hybridization, ligation, and PCR amplification in one tube, the amplicons were detected using albumin (ALB) and SMN1 probes targeted against crRNAs. Theoretically, for normal individuals carrying two copies of *SMN1* and *ALB*, the final ratio of increment in fluorescence value of *SMN1* to that of *ALB* is close to 1. In SMA carriers, the ratio is 0.5. In spinal muscular atrophy (SMA) patients, the ratio is 0. (B) The change curves of fluorescence value obtained from normal individuals, SMA carriers, and SMA patients using the one‐tube BCDetection method. *Δ*, represents subtracted the background fluorescence. Error bars indicate mean ± SEM; *n* = 3. (C) Comparison of the ratio of increment in *SMN1* fluorescence value to *ALB* fluorescence value in normal individuals, SMA carriers, and SMA patients detected using BCDetection. Error bars indicate means ± SD; *n* = 20. ****, *p* < 0.0001.

Given BCDetection can translate multiple target sequences to corresponding probe‐ligated products whose lengths are similar and with the same primers for amplification, we further optimized the quantification detection system to simplify the operation steps and eliminate the difference in the initial template quantity required between the targeted and internal reference genes. We redesigned the SMN1‐probe (SMN‐P1/P2) that targeted *SMN1* c.840 and ALB‐probe (ALB‐P3/P2) that targeted *ALB* to facilitate the ligation and amplification of exon 7 of *SMN1* and *ALB* in one tube. The products were detected using SMN‐crRNA for *SMN1* and ALB‐crRNA for *ALB* (Figure 6A). We extracted gDNA from normal individuals, SMA carriers, and patients with SMA. Consistent with the two‐tube method, the fluorescence value curves of *SMN1* exon 7 and *ALB* almost overlapped in the normal participants. In SMA carriers, the fluorescence curves of *SMN1* exon 7 were always lower than those of *ALB*. For SMA patients, the fluorescence curve of *SMN1* exon 7 was near the baseline (Figure 6B). Detection was performed using one tube (one‐tube method) to analyze 60 clinical samples. The ratio of fluorescence values in normal individuals (1.02 ± 0.09), SMA carriers (0.67 ± 0.06), and SMA patients (0.06 ± 0.02) (Figure 6C). These results demonstrated that BCDetection could significantly distinguish CNV in one tube.

## 3 DISCUSSION

In this study, we established PCDetection and BCDetection systems for simultaneous detection of multiple targets without PAM sequence restriction and verified the feasibility to detect multiple mutation sites of β‐thalassemia carriers in a single reaction. Various reaction conditions were optimized, such as ligation cycles and PCR cycles. Moreover, a BCDetection system for accurate quantification of copy number was established. We evaluated the feasibility for distinguishing the SMA patients, SMA carriers, and normal individuals and demonstrated that BCDetection could significantly distinguish them in one tube.

The simultaneous detection of multiple targets is one of the most important merits of CRISPR/Cas. Gootenberg et al.^15^ established a multiplex detection platform, SHERLOCKv2, which can detect four targets in a single reaction. However, this platform relies on different reporters, crRNAs, four kinds of Cas enzymes, and restriction by the PAM/PFS.^15^ Simultaneous detection of multiple targets provide more important disease‐related information than single‐channel detection. Here, multiple targets are rapidly and efficiently detected simultaneously by PCDetection and BCDetection.

Traditionally, individuals with decreased mean corpuscular volume and mean corpuscular hemoglobin but increased HbA2 could be suspected as β‐thalassemia carriers.^19^ Subsequently, the positive individuals undergo molecular testing for genotype determination.^20^ However, this screening technique cannot identify carriers with negative hematological phenotypes. There are 129‐point mutations and 16 deletion mutations that have been identified in the Chinese β‐thalassemia population, of which eight mutations are found in more than 95% of β‐thalassemia carriers.^21^ PCDetection and BCDetection provide another strategy for the straightforward detection of pathogenic variants in β‐thalassemia carrier screening via the detection of multiple mutation sites in a single reaction, which holds the methodological advantage of multiplex assay, high efficacy, and low costs.

Although several CNV detection techniques, such as multiplex ligation‐dependent probe amplification (MLPA),^22^, ^23^ digital PCR^24^, ^25^, ^26^ chromosome microarray analysis,^27^ and CNV sequencing^28^, ^29^ have been developed, these methods have a limitation that is the need for experienced technicians to conduct tests on sophisticated instruments and process data using professional software. Using combination of digital PCR,^16^ volumetric bar‐chart chips,^30^ or gold electrodes,^31^ CRISPR/Cas has been widely used for quantitative detection. However, quantification of CNV remains challenging. To the best of our knowledge, for the first time, we have established an accurate and simple CNV quantitative detection system based on the CRISPR/Cas method.

In summary, we refined Cas12a‐based detection to ensure PAM‐free, quantitative, and multiplexed readouts, enabling comprehensive nucleic acid detection. With efficient transformation and amplification of hybridization signals to fluorescence signals, BCDetection not only enables detection of nucleic acid targets without intrinsic PAM restriction, but also expands application potential from qualitative detection to quantitative and multiplex detection in one tube. This proposed method can be used for multiplex genotype profiles that associate with numerous diseases. Moreover, with the help of the internal reference gene, BCDetection enables unbiased and accurate quantification of copy number, providing an opportunity for the quantitative detection of CNV of numerous genetic diseases or copy number of pathogens. Given the versatility, programmability, and flexibility of the CRISPR/Cas12a platform, BCDetection can be used for multiple purposes. In the future, a nucleic acid detection platform for rapid, multiple, and visual measurements of different targets combined BCDetection with isothermal amplification and colloidal gold strips can be developed, which could have significant implications for genetic analysis and individualized disease diagnosis.

## 4 MATERIALS AND METHODS

### 4.1 Sample source

The study samples were obtained from Hunan Jiahui Genetics Hospital, and the detailed information of the samples is summarized in Tables S1 and S2 (in the supplementary materials). The gDNA was extracted from peripheral blood of the participants using the conventional phenol–chloroform method.

### 4.2 Primers, probes, and crRNAs

PCR primers, probes, and crRNAs listed in Table S3 were synthesized by Sangon Biotech.

For PCDetection, the primer HBB‐28‐F included the universal recognition sequence of crRNA and PAM, and the 3′‐end of the primer contained the mutated base that was modified with a locked nucleic acid. The targeted sequence could be amplified with HBB‐28‐F and HBB‐28‐R when the gDNA contained a variant of *HBB*:c.‐78A>G. The design of the primers CD41‐42‐F/R and IVS‐II‐654‐F/R was similar to that of the primers HBB‐28‐F/R.

For BCDetection, probe 1 (P1) contained a universal primer F sequence for amplification, a universal recognition sequence of crRNA and PAM, and the 3′‐end sequence that could bind to the complementary target sequence (the sequence for ligation and recognition). Probe 2 (P2) included a universal primer R‐binding site for amplification and complementary binding sequences of the target (the sequence for ligation and recognition). When the gDNA contains complementary sequences that can bind to the sequence in P1 for complete ligation and recognition, P1 and 2 can recognize, bind to the target sequence, and ligate into single‐stranded DNA, providing a template for amplification and Cas12a detection.

### 4.3 PCDetection for detecting three β‐thalassemia pathogenic mutations

First, we detected three β‐thalassemia mutations, c.‐78A>G (‐28), CD41‐42, and IVS‐II‐654, using PCDetection. PCR was performed using the corresponding primers F/R, with a 20 μL reaction volume containing 10 μL of 2× Premix Ex Taq Hot Start (Takara), 7 μL of ddH_2_O, 1 μL of primer F (10 μM), 1 μL of primer R (10 μM), and 1 μL of gDNA (10 ng/μL) under the following thermocycling conditions: 95°C for 5 min; followed by 30 cycles of 95°C for 30 s, 63°C for 30 s, and 72°C for 30 s; and elongation at 72°C for 5 min. The PCR products were used for Cas12a detection. The Cas12a assay mixture contained 2 μL of 10× Buffer 2.1 (New England Biolabs), 100 nM Lba Cas12a, 50 nM crRNA, 500 nM FQ probe (Sangon Biotech), and 2 μL of PCR products, and the total volume was made up to 20 μL using nuclease‐free water. The reaction solution was incubated at 37°C for 30 min, and the FAM fluorescence signal was measured every minute.

Next, we detected three β‐thalassemia mutations in a single reaction. The mixed primer F comprised 10 μM of HBB‐28‐F, 10 μM of CD41‐42‐F, and 10 μM of IVSII‐654‐F. And the mixed primer R included 10 μM of HBB‐28‐R, 10 μM of CD41‐42‐R, and 10 μM of IVSII‐654‐R was mixed. PCR was performed using mixed primers F/R, with a 20 μL reaction volume containing 10 μL of 2× Premix Ex Taq Hot Start (Takara), 7 μL of ddH_2_O, 1 μL of mixed primer F, 1 μL of mixed primer R, and 1 μL of gDNA (10 ng/μL) under the following thermocycling conditions: 95°C for 5 min; followed by 30 cycles of 95°C for 30 s, 63°C for 30 s, and 72°C for 30 s; and elongation at 72°C for 5 min. The PCR products were used for Cas12a detection. The Cas12a assay was performed using the aforementioned procedure.

### 4.4 BCDetection employing a fluorescence probe

BCDetection was established, and fluorescence signals were detected to optimize the number of ligation cycles and PCR amplification cycles. The 25 μL ligation system contained 2.5 μL of 10× HiFi Taq DNA Ligase Buffer (New England Biolabs), 0.5 μL of HiFi Taq DNA Ligase (New England Biolabs), 17 μL of ddH_2_O, 1.25 μL of SMN‐P1 targeted against c.840 of *SMN1* (100 fM), 1.25 μL of SMN‐P2 targeted against c.840 of *SMN1* (100 fM), and 2.5 μL of gDNA (10 ng/μL); the thermocycling conditions used were as follows: 95°C for 10 min, followed by various cycles (5, 10, and 20 cycles) of 95°C for 30 s and 60°C for 1 min, and then incubation at 10°C. The ligation products were used as templates for PCR.

PCR was performed using corresponding primers F/R, with a 20 μL reaction volume containing 10 μL of 2× Premix Ex Taq Hot Start (Takara), 4 μL of ddH_2_O, 1 μL of primer F (10 μM), 1 μL of primer R (10 μM), and 4 μL of ligation products; the thermocycling conditions were as follows: 95°C for 5 min; followed by different cycles (25, 30, and 35 cycles) of 95°C for 30 s, 59°C for 30 s, and 72°C for 30 s; and then elongation at 72°C for 5 min.

The PCR products were used for Cas12a detection. The Cas12a assay mixture contained 2 μL of 10× Buffer 2.1 (New England Biolabs), 100 nM Lba Cas12a (New England Biolabs), 50 nM crRNA, 500 nM FQ probe (Sangon Biotech), and 2 μL of PCR products, and the total volume was made up to 20 μL using nuclease‐free water. The reaction solution was incubated at 37°C for 30 min, and the FAM fluorescence signal was measured every minute.

### 4.5 BCDetection for detecting three β‐thalassemia pathogenic mutations

P1 for three β‐thalassemia mutations was designed and synthesized. The probe targeting HBB‐28, HBB CD41‐42, and HBB‐IVSII‐654 were HBB‐28‐P1/P2, CD41‐42‐P1/P2, and IVSII‐654‐P1/P2, respectively. Separate ligation system was used for HBB‐28, HBB CD41‐42, and HBB‐IVSII‐654 for the same sample, and the PCR amplification was performed separately. The ligation cycle was performed for five cycles, and PCR amplification was performed for 30 cycles. The aforementioned conditions were used for ligation, PCR amplification, and Cas12a detection.

Three β‐thalassemia mutations were detected in a single reaction with BCDetection. Mixed P1 included 100 fM of HBB‐28‐P1, 100 fM of CD41‐42‐P1, and 100 fM of IVSII‐654‐P1. Then, 100 fM of HBB‐28‐P2, 100 fM of CD41‐42‐P2, and 10 μM of IVSII‐654‐P2 were mixed. Ligation was performed using mixed Probe1/2, with the 20 μL reaction volume containing 2.5 μL of 10× HiFi Taq DNA Ligase Buffer, 0.5 μL of HiFi Taq DNA Ligase, 17 μL of ddH_2_O, 3.75 μL of the mixed P1, 3.75 μL of the mixed P2, and 2.5 μL of gDNA (10 ng/μL). The aforementioned conditions were used for ligation, PCR amplification, and Cas12a detection.

### 4.6 Quantitative detection performance of BCDetection

To determine the quantitative detection performance of BCDetection, DNA samples from normal participants were serially diluted to 200, 100, 50, 25, 12.5, 6.25, 3.125, 1.5625, and 0.78125 ng/μL and were tested using BCDetection. The fluorescence signal was measured every minute, and the correlation between fluorescence and gDNA concentration was analyzed using linear regression.

### 4.7 BCDetection in two tube

The probes targeting *SMN1* exon 7 (SMN‐P1/P2) and albumin gene (*ALB*) (ALB‐P1/P2) were used for quantitative detection. gDNA isolated from the peripheral blood of 20 normal individuals, 20 spinal muscular atrophy (SMA) carriers, and 20 SMA patients were used. The copy number of *SMN1* exon 7 in all the samples was determined using MLPA. Due to the same crRNA sequence for SMN‐P1 and ALB‐P1, the ligation was performed separately for *SMN1* and *ALB* for the same sample, and the PCR amplification system also required two reaction mixtures for *SMN1* and *ALB* for the same sample. The aforementioned conditions were used for ligation, PCR amplification, and Cas12a detection.

### 4.8 BCDetection assay in one tube

The probes targeting *SMN1* exon 7 (SMN‐P1/P2) and *ALB* (ALB‐P3/P2) were used. gDNA samples from 20 normal individuals, 20 SMA carriers, and 20 SMA patients were used as templates. As SMN‐crRNA was used for SMN‐P1 and ALB‐crRNA was used for ALB‐P3, the ligation for *SMN1* and *ALB* could be performed in the same mixture; hence, the PCR amplification system needed only one reaction mixture for *SMN1* and *ALB* for the same sample.

The 25 μL ligation system contained 2.5 μL of 10× HiFi Taq DNA Ligase Buffer, 0.5 μL of HiFi Taq DNA Ligase, 14.5 μL of ddH_2_O, 1.25 μL of SMN‐P1 (100 fM), 1.25 μL of SMN‐ P2 (100 fM), 1.25 μL of ALB‐P3 (100 fM), 1.25 μL of ALB‐P2 (100 fM), and 2.5 μL of gDNA (10 ng/μL); the thermocycling conditions were as follows: 95°C for 10 min, followed by 5 cycles of 95°C for 30 s and 60°C for 1 min, and then incubation at 10°C. The ligation products were used as templates for PCR.

PCR was performed using the corresponding primers probe‐F/probe‐R, with a 20 μL reaction volume containing 10 μL of 2× Premix Ex Taq Hot Start (Takara), 4 μL of ddH_2_O, 1 μL of primer probe‐F (10 μM), 1 μL of primer probe‐R (10 μM), and 4 μL of ligation product. The thermocycling conditions were as follows: 95°C for 5 min; followed by 30 cycles of 95°C for 30 s, 59°C for 30 s, and 72°C for 30 s; and elongation at 72°C for 5 min.

The PCR products were used for Cas12a detection. For *SMN1* detection, the Cas12a assay mixture contained 2 μL of 10× Buffer 2.1 (New England Biolabs), 100 nM Lba Cas12a, 50 nM SMN‐crRNA, 500 nM FQ probe (Sangon Biotech), and 2 μL of PCR products, and the total volume was made up to 20 μL using nuclease‐free water. For ALB detection, the Cas12a assay mixture contained 2 μL of 10× buffer 2.1 (New England Biolabs), 100 nM Lba Cas12a, 50 nM ALB‐crRNA, 500 nM FQ probe (Sangon Biotech), 2 μL of PCR products, and the total volume was made up to 20 μL using nuclease‐free water. The reaction solution was incubated at 37°C for 30 min, and the FAM fluorescence signal was measured every minute.

### 4.9 Statistical analyses

Data were analyzed using GraphPad Prism 8 (GraphPad Software Inc., San Diego, CA, USA). Data between two groups were compared using the Student's *t*‐test, whereas data among multiple groups were compared using one‐way analysis of variance.

## AUTHOR CONTRIBUTION

M. Z., L. W., and D. L. conceived the project and designed the experiments. M. Z. and Z. H. wrote the original draft. C. Z., Z. H., and D. L. revised the manuscript. C. Z., M. C., M. L., and Z. L. performed the experiments. M. Z. and C. Z. analyzed and interpreted the experimental data. L. W. and D. L. supervised the study. All authors have read and approved the final manuscript.

## CONFLICT OF INTEREST STATEMENT

The authors declare that they have no known competing financial interests or personal relationships that could have appeared to influence the work reported in this paper. The study samples were obtained from Hunan Jiahui Genetics Hospital, but it has no potential relevant financial or nonfinancial interests to disclose.

## ETHICS STATEMENT

This study was approved by the Ethics Committee of the School of Life Sciences, Central South University (No. 2019‐1‐16, 11 March 2019). Written informed consent was obtained from all participants.

## Supporting information

Supporting Information

## Data Availability

The data that support the findings of this study are available from the corresponding author upon reasonable request.
