# Supplementary material for: A protospacer adjacent motif‐free, multiplexed, and quantitative nucleic acid detection platform with barcode‐based Cas12a activity
Source: MedComm (2020). 2023 Jul 2;4(4):e310. doi: 10.1002/mco2.310 (PMC10315165; doi:10.1002/mco2.310)
Supplement: Supplementary file 1 — Supporting Information [file MCO2-4-e310-s001.docx]

**A PAM-free, Multiplexed, and Quantitative Nucleic Acid Detection Platform with Barcode-Based Cas12a Activity**

Miaojin Zhou ^1,#^, Chunhua Zhang ^1,2,#^, Miaomiao Chen ^1, #^, Zhiqing Hu ^1^, Menglin Li ^1^, Zhuo Li ^1^, Lingqian Wu ^1,*^, Desheng Liang ^1,*^

^1^ Center for Medical Genetics & Hunan Key Laboratory of Medical Genetics, School of Life Sciences, Central South University, Changsha, Hunan 410078, China.

^2^ Yunnan Maternal and Child Health Care Hospital, Kunming, Yunnan 650051, China.

^#^ These authors contributed equally.

* Corresponding Author:

Lingqian Wu, E-mail: wulingqian@sklmg.edu.cn.

Desheng Liang, E-mail: liangdesheng@sklmg.edu.cn.

**Running title**: Nucleic Acid Detection via BCDetection

Supplementary Table 1. Information of samples used in β-thalassaemia assay.

| Sample source | Cohort | Genotype | Number of samples |
| --- | --- | --- | --- |
| Residual DNA | β-thalassaemia carrier | *HBB*:c.-78A>G | 3 |
|  |  | *HBB*:c.126_129delCTTT | 3 |
|  |  | *HBB*:c.316-197C>T | 3 |
| Residual DNA | Normal individuals | No deletion | 3 |

Supplementary Table 2. Information of samples used in SMA assay.

| Sample source | Cohort | Genotype of *SMN1* exon 7 | Number of samples |
| --- | --- | --- | --- |
| Residual DNA | SMA patients | Homozygous deletion | 20 |
| Residual DNA | SMA carriers | Heterozygous deletion | 20 |
| Residual DNA | Normal individuals | No deletion | 20 |

Supplementary Table 3. Detailed sequence of primers, crRNAs and probes.

| Use for | Name | Sequence |
| --- | --- | --- |
| PCDetection | crRNA | 5’-UAAUUUCUACUAAGUGUAGAUGCACAAUGAAGU GGGUAACCUUU-3’ |
|  | ALB-crRNA | 5’-UAAUUUCUACUAAGUGUAGAUGUCUGUACUACA CACAGUACUGC-3’ |
|  | HBB-28-F | 5’-TGCTCTGTCTGCATGGGTTTGGCACAATGAAGTGG GTAACCTTTCAGGGCTGGGCATA**G**-3’ |
|  | HBB -28-R | 5’-CACCATGGTGTCTGTTTGAG-3’ |
|  | CD41-42-F | 5’-TGCTCTGTCTGCATGGGTTTGGCACAATGAAGTGG GTAACCTTTCAGTGGACCCAGAGGTTGA**G**-3’ |
|  | CD41-42-R | 5’-TGCCCATAACAGCATCAGG-3’ |
|  | IVS-II-654-F | 5’-TGCTCTGTCTGCATGGGTTTGGCACAATGAAGTGG GTAACCTTTTGATAATTTCTGGGTTAAGG**T**-3’ |
|  | IVS-II-654-R | 5’-GCAGAATGGTAGCTGGATTG-3’ |
|  | fluorophore-quencher probe | 5’-FAM-TTTTTTTTTTTT-MGB-3’ |
| BCDetection | SMN-P1 | 5’-TGCTCTGTCTGCATGGGTTTGGCACGATGAAGTGG GTAACCTTTCCTTCCTTCTTTTTGATTTTGTCTG-3’ |
|  | SMN-P2 | 5’-P-AAACCCTGTAAGGAAAATAAAGGAACATGTCGA AGCTCATGGGC-3’ |
|  | ALB-P1 | 5’-TGCTCTGTCTGCATGGGTTTGGCACGATGAAGTGG GTAACCTTTGTGGTCCTGAACCAGTTATG-3’ |
|  | ALB-P3 | 5’-TGCTCTGTCTGCATGGGTTTGGTCTGTACTACACAC AGTACTGCGTGGTCCTGAACCAGTTATG-3’ |
|  | ALB-P2 | 5’-P-TGTGTTGCATGAGAAAACGCGATGTCGAAGCTC ATGGGC-3’ |
|  | probe-F | 5’-TGCTCTGTCTGCATGGGTT-3’ |
|  | probe-R | 5’-GCCCATGAGCTTCGACAT-3’ |
|  | HBB-28-P1 | 5’-TGCTCTGTCTGCATGGGTTTGGTCTATACTACACAC AGTACTGCCCAGGGCTGGGCATAG-3’ |
|  | HBB-28-P2 | 5’-P-AAGTCAGGGCAGAGCCACATGTCGAAGCTCAT GGGC-3’ |
|  | CD41-42-P1 | 5’-TGCTCTGTCTGCATGGGTTTGGTCTATACTACACA CAGTACTGCCCCTTGGACCCAGAGGTT-3’ |
|  | CD41-42-P2 | 5’- P-GAGTCCTTTGGGGATCTGTCCCATGTCGAAGCTC ATGGGC-3’ |
|  | IVSII-654-P1 | 5’-TGCTCTGTCTGCATGGGTTTGGTCTATACTACACA CAGTACTGCAACAGTGATAATTTCTGGGTTAAGGT-3’ |
|  | IVSII-654-P2 | 5’- P-AATAGCAATATCTCTGCATATAAATATTTCTGAT GTCGAAGCTCATGGGC-3’ |
|  | SMN-crRNA | 5’-UAAUUUCUACUAAGUGUAGAUGCACAAUGAAGU GGGUAACCUUU-3’ |
|  | ALB-crRNA | 5’-UAAUUUCUACUAAGUGUAGAUGUCUGUACUACA CACAGUACUGC-3’ |
|  | fluorophore-quencher probe | 5’-FAM-TTTTTTTTTTTT-MGB-3’ |

Bolded and underlined letters indicated locked nucleic acid (LNA).

Letter P indicated the primers contained a 5’-terminal phosphate moiety.
